# Characterizing the Diversity of the CDR-H3 Loop Conformational Ensembles in Relationship to Antibody Binding Properties

**DOI:** 10.3389/fimmu.2018.03065

**Published:** 2019-01-07

**Authors:** Monica L. Fernández-Quintero, Johannes R. Loeffler, Johannes Kraml, Ursula Kahler, Anna S. Kamenik, Klaus R. Liedl

**Affiliations:** Center for Molecular Biosciences Innsbruck (CMBI), Institute of General, Inorganic and Theoretical Chemistry, University of Innsbruck, Innsbruck, Austria

**Keywords:** antibodies, CDR-H3 loop, affinity maturation, molecular dynamics, enhanced sampling, conformational selection, markov-state model

## Abstract

We present an approach to assess antibody CDR-H3 loops according to their dynamic properties using molecular dynamics simulations. We selected six antibodies in three pairs differing substantially in their individual promiscuity respectively specificity. For two pairs of antibodies crystal structures are available in different states of maturation and used as starting structures for the analyses. For a third pair we chose two antibody CDR sequences obtained from a synthetic library and predicted the respective structures. For all three pairs of antibodies we performed metadynamics simulations to overcome the limitations in conformational sampling imposed by high energy barriers. Additionally, we used classic molecular dynamics simulations to describe nano- to microsecond flexibility and to estimate up to millisecond kinetics of captured conformational transitions. The methodology represents the antibodies as conformational ensembles and allows comprehensive analysis of structural diversity, thermodynamics of conformations and kinetics of structural transitions. Referring to the concept of conformational selection we investigated the link between promiscuity and flexibility of the antibodies' binding interfaces. The obtained detailed characterization of the binding interface clearly indicates a link between structural flexibility and binding promiscuity for this set of antibodies.

## Introduction

Antibodies have emerged as essential therapeutic agents in the treatment of cancer and various other diseases (1). The importance of therapeutic antibodies for the pharmaceutical industry has increased substantially in the past decade (2). A key challenge in antibody design is tailoring their binding specificity on the one hand to allow cross-species toxicity tests and on the other hand to avoid off-target effects (3). The specificity of an antibody, mainly influenced by the complementary determining region (CDR), plays a key role in antigen recognition and binding processes (4). The CDR is composed of six hypervariable loops, three formed by each chain, that shape the paratope, i.e., the antigen binding site of the antibody. Five of the six loops usually adopt well-characterized canonical conformations, which facilitates reliable structure prediction based on sequence information (5–8). Yet, the CDR-H3 loop shows substantial variability in sequence and structure and hence cannot be described by a canonical structure model (9, 10). Even compared to other protein loop structures, the CDR-H3 clearly stands out with its significantly higher structural diversity (11). Thus, computational modeling of the CDR-H3 loop is particularly challenging and optimized strategies are required to predict accurate structures (9, 12). A clear characterization of structure and dynamics of an antibody is essential to understand the antigen binding process, the involved conformational changes and the associated biological implications (13). Historically, protein-protein interactions such as antibody-antigen binding were assumed to follow the “lock and key”(14) mechanism suggesting a rigid complementary paratope and a rigid antigen (15). However, this “lock and key” hypothesis has been challenged by the “induced fit”(16) and the “conformational selection”(17) concepts. While the “induced fit” binding paradigm argues for structural rearrangements as response to the binding process, “conformational selection” follows the idea of an ensemble of pre-existing conformational states with varying probabilities from which a binding competent state is selected (17–19).

Both mechanisms have been discussed in current literature to explain the binding preferences of polyreactive antibodies (19, 20). Regardless of the underlying binding mechanism, it has been shown that the binding site of polyreactive monoclonal antibodies, which bind with low affinity to various structurally unrelated antigens, is inherently more flexible compared to high-affinity antibodies (21, 22). Depending on the antigen present, polyreactive antibodies have been observed to display varying conformations of their binding site, reflected by a higher conformational diversity in the CDR (4). Especially the CDR-H3 loop is known to have a substantial impact on the shape of the paratope and thus strongly influences antigen binding (23). However, the role of the CDR-H3 loop for specificity in antigen-recognition is still debated (24).

The correlation between rigidification and enhanced specificity is often discussed in terms of conformational selection (17, 25). A direct connection between promiscuity and flexibility can be observed in the affinity maturation process (26). In this process antibodies with increased affinity for antigens are produced by activated B-cells during the immune response. Repeated exposure of the same antigen leads to mutations in the sequence that predominantly cause a rigidification of the antigen binding site (4, 26). However, in other cases it has been reported that affinity maturation does not necessarily lead to rigidification of the CDR-H3 loop (27).

In this study we test the hypothesis that promiscuity might arise from a multitude of weakly populated conformations, each of which is able to bind different binding partners. Rigidification shifts the probability toward a small number of states and hence reduces the amount of possible binding partners. This would mean that affinity maturation disfavors sequences that are intrinsically flexible and promotes sequences that lead to a single conformation (28). We present three examples with and without prior structural information where affinity maturation/reduced promiscuity leads to a significant rigidification of the CDR-H3 loop (29–31). We chose the antibody pairs with the focus on the availability of experimental data (information on binding properties and structural information) and on the CDR-H3 loop length. We aimed for different lengths of the CDR-H3 loop, nevertheless preferring rather short loops (<15 residues). The first pair of antibodies is the ferrochelatase antibody 7G12, which catalyzes the porphyrin metalation. In complex with mesoporphyrin the 7G12 antibody forms the Michaelis complex. The affinity matured 7G12 antibody compared with the naïve antibody shows the molecular mechanism how the immune system processes the binding energy to catalyze this metalation reaction (29). The second pair of antibodies shows antibodies in different stages of affinity maturation, both evolved from the same germline precursor to bind the chromophoric antigen 8-methoxypyrene-1,2,6-trisulfonate (MTPS). The antibodies have been characterized by their sequence, molecular recognition and with three-pulse photon echo peak shift spectroscopy to identify the influence of mutations on plasticity, specificity and anelasticity (30). The third antibody pair was chosen from a synthetic library. Birtalan et al. (31) analyzed the contributions of four amino acids Arg, Tyr, Gly, and Ser to affinity and specificity in antigen recognition using synthetic antibody libraries without providing further structural characterization. The available antibodies were tested against a set of eight antigens and we chose a pair of sequences with the same CDR-H3 loop length (10 residues) showing substantial differences in their affinities to the eight antigens.

## Methods

### First Antibody Pair: Affinity Maturation of Germline Antibody 7G12

Crystal structures (PDB codes: 1N7M, 1NGZ, 1NGY, 1NGW) (29) for the germline antibody 7G12 and the affinity-matured antibody 7G12 are available in the Protein Data Bank (PDB) (32) both with and without the antigen (N-methylmesoporphyrine) bound. The structures of the antibody variable domains (Fvs) are illustrated in Figure S1. The main structural differences are located in the CDR-H3 loop of the naïve antibody between the bound and the free state, which show a Cα-RMSD of 2.3 Å (Figures S1,S5). The sequences of the naïve and the matured antibody Fv differ in six amino acid residues, three mutations in the heavy and the light chain, each. The only mutation in the CDR-H3 loop is S97M.

### Second Antibody Pair: Affinity Maturation of 6C8 to 8B10

The second pair of systems are the initially matured antibody 6C8 and the further affinity-matured antibody 8B10 (30). Crystal structures are available in the PDB (PDB codes: 4NJA and 4NJ9). The initially matured antibody 6C8 is similar to the germline and differs only in the single mutation I30N in V_L_. The sequences of the Fv of the initially matured 6C8 and of the further matured antibody 8B10 differ in five amino acid residue mutations, two in the light chain and three in the heavy chain. The affinity-matured 8B10 contains the mutation I100S in the CDR-H3 loop. This additional serine in the CDR-H3 loop forms water-mediated hydrogen-bonds with the CDR-L3 loop. The crystal structures (Figure S6) do not show significant differences in the backbone conformations (Cα-RMSD 1.4 Å), however the sidechains of the CDR-H3 loop are more stabilized due to the additional hydrogen bonds in the matured antibody (30).

### Third Antibody Pair: Specific Antibody Fab 246 and Promiscuous Antibody Fab 249

To predict the structure of the Fv region of the CDR-sequences (Fab 246, Fab 249) (31), the program RosettaAntibody (33–35) was applied. We assume that the structural modeling works reliably for five of the six CDR loop regions, i.e., those that can be characterized by canonical structures (3). The CDR loops that served as templates for the modeling are listed in Table S1. For the diversification of the CDR-H3 loop we used the KIC algorithm implemented in Rosetta to generate 100 loop structures (36, 37). The resulting 100 loop conformations were clustered using a hierarchical clustering algorithm as implemented in cpptraj applying a distance cutoff criterion of 3.0 Å (38, 39). The applied clustering scheme resulted in 4 clusters. Four structural models of each system with structural differences in the CDR-H3 loop were used as starting structures for metadynamics simulations (Figure S11).

### Combined Simulation and Analysis Protocol for All Six Antibodies

For the two pairs of antibodies where crystal structures were available, those were used as starting structures for metadynamics simulations. All structures were prepared in MOE (Molecular Operating Environment) (40) using the Protonate3D (41) tool. The C-termini of the antibodies were capped with N-Methylamine (NME). With the tleap tool of the AmberTools16 (38) package, the two systems were soaked into cubic water boxes of TIP3P water molecules (42) with a minimum wall distance to the protein of 10 Å. Parameters for all antibody simulations were derived from the AMBER force field 14SB (43). Each system was carefully equilibrated using a multistep equilibration protocol (44). To achieve an extensive but efficient exploration of the conformational space, well-tempered metadynamics simulations were performed using GROMACS (45), i.e., plumed 2 (46) software package. In metadynamics simulations a history-dependent bias potential is built based on Gaussian functions, which are deposited on the potential energy surface at already sampled conformations (47). This leads to an accelerated sampling allowing the system to escape deep energy minima. Well-tempered metadynamics (48) adapts the height of the Gaussian functions with simulation time. Various collective variables (CV) have been tested to achieve a better description of the conformational space. The most efficient CVs for our systems were found to be linear combinations of sine and cosine of the ψ torsion angles (49) of the CDR-H3 and CDR-L3 loops, which were calculated with functions MATHEVAL and COMBINE implemented in plumed 2 (46). As discussed previously, the ψ torsion angle captures conformational transitions comprehensively (50–52). The decision to include the CDR-L3 loop is based on previously observed structural correlation of the CDR-L3 and CDR-H3 loop (53). The height of the Gaussian was determined according to minimal distortion of the antibody systems, resulting in 10.0 kcal/mol for the antibodies with structural information and 2.0 kcal/mol for the Fab 246 and Fab 249. Gaussian deposition occurred every 1,000 steps and a biasfactor of 10 was used. For all 6 antibodies we collected for each starting structure 1 μs by metadynamics simulations. The resulting trajectories were aligned to the entire Fv and clustered in cpptraj (38, 39) using the average-linkage hierarchical clustering algorithm. The Cα-RMSD of the CDR-H3 loop was used as distance metric and the same cutoff criterion was applied for each pair of antibodies. The choice of the distance cutoff is optimized to obtain a broad cluster distribution within Principle Component Analysis (PCA) space for each system. For the Fab 246 and Fab 249 antibody pair we chose 200 cluster representatives each to compensate for the uncertainty introduced by using modeled structures. The resulting cluster representatives for all systems were equilibrated and simulated for 100 ns using classic MD as implemented in the AMBER16 simulation package (38). Molecular dynamics simulations were performed in an NpT ensemble using pmemd.cuda (38, 54, 55). Bonds involving hydrogen atoms were restrained by applying the SHAKE (56) algorithm, allowing a time step of 2.0 fs. Atmospheric pressure of the system was preserved by weak coupling to an external bath using the Berendsen algorithm (57). The Langevin thermostat (58) was used to maintain the temperature at 300 K during simulations.

The obtained MD trajectories for each system were analyzed with PCA and the time-lagged independent component analysis (tICA) (59) of the Cα CDR-H3 loop atoms using the python library PyEMMA 2 (60) and employing a lag time of 5 ns. tICA can be used as a dimensionality reduction method and is a technique to find the slowest-relaxing degrees of freedom. Compared to PCA, which leads to high-variance linear combinations of the input degrees of freedom, tICA shows high-autocorrelation linear combinations of the input degrees of freedom (61, 62). The tICA space was used for clustering to generate microstates that build the basis for a MSM. The aim of the Markov-state models is to define kinetically relevant states, to estimate the transition times between them and to quantify the probability of the states (63). Thus, kinetics were estimated by constructing a Markov-state model (63) employing a lag time of 5 ns using PyEMMA 2. We chose the lag time according to the implied timescale plot, which shows an approximately constant behavior of the estimated timescales at lag times over 5 ns (64, 65). We used k-means clustering (60) to define 200 microstates and the PCCA+ algorithm (66) to calculate macrostates and estimate their according representative structures. PCCA+ is a spectral clustering method, which discretizes the sampled conformational space based on the eigenvectors of the transition matrix.

## Results

### Affinity Maturation of Germline Antibody 7G12

**Figure 1:**
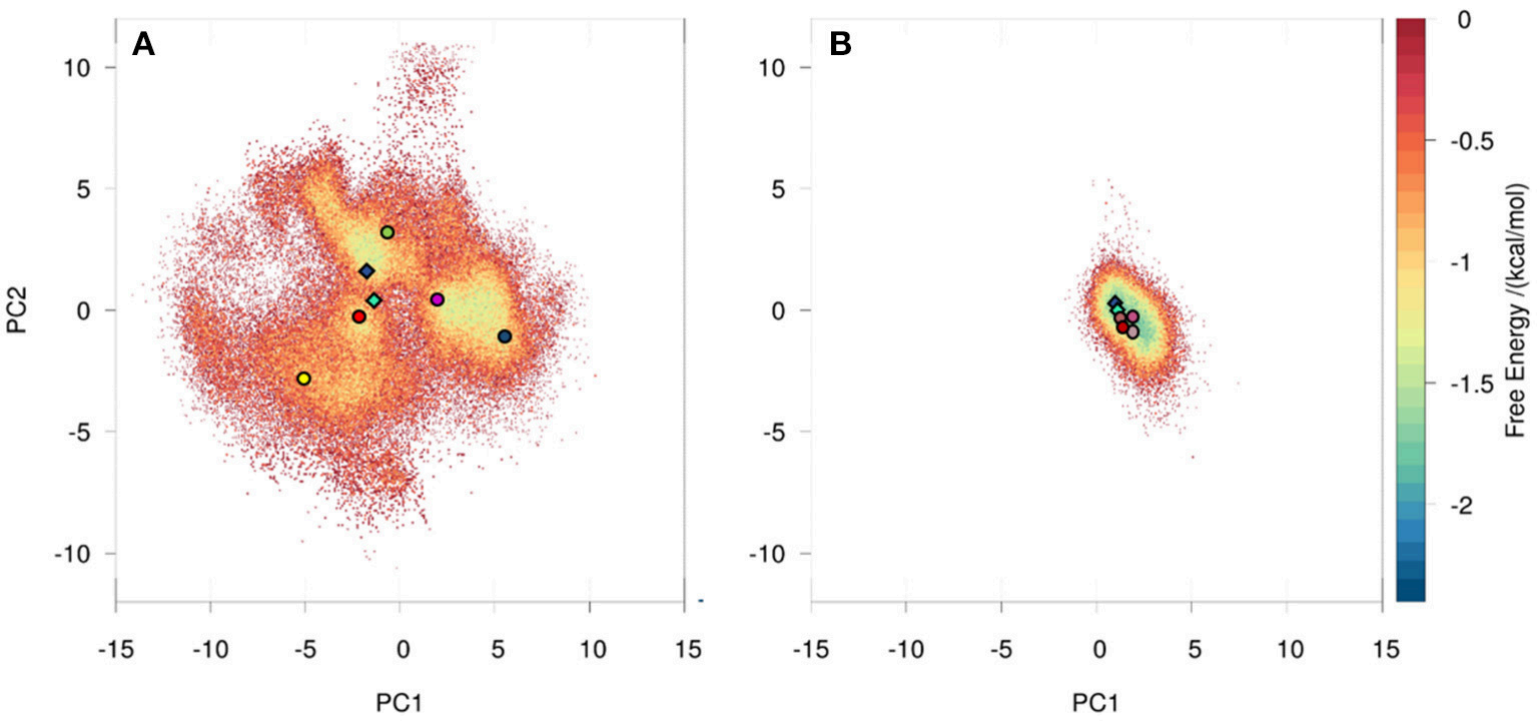
Conformational space of the naïve **(A)** and the matured **(B)** 7G12 antibody with the projection of the crystal structures as diamonds (bound state: dark blue, free state: cyan). The variances for the PC1 and PC2 are 45 and 20% respectively. Both 10 μs trajectories are projected onto the combined PCA space, resulting in one distinct and narrower free energy basin for the matured and a shallow and broad free energy surface for the naïve antibody. Projections of representative structures for each Markov state are depicted as circles color-coded according to the Markov-state model shown in Figure 2.

We observed a broader conformational ensemble for the naïve antibody. To visualize the decreased flexibility of the matured antibody compared to the naïve antibody a principle component analysis (PCA) of the CDR-H3 loop was performed (Figure S2). As described in the methods section, the metadynamics simulations of the bound and free starting structure were combined and the naïve and matured antibodies were clustered separately with a hierarchical average linkage clustering algorithm (38). The distance criterion was defined with the aim of having representative structures distributed over the entire sampled conformational space, covering all free energy minima as well as transition regions in the PCA space (Figure S2). This strategy resulted in 102 clusters for the naïve antibody, while it led to only 7 clusters for the matured antibody with a distance cutoff criterion of 1 Å for both cases. The resulting cluster representatives were used as starting structures to seed classic MD simulations of 100 ns length. To obtain comparable overall simulation times for both antibodies, for each of the 7 cluster structures of the matured antibody 12 runs of 100 ns MD simulations with different starting velocities were performed. We performed PCA combining the coordinates of both systems (Figure 1) and estimated the probability resulting from the accumulated 10 μs of classic MD simulations. The analysis clearly depicts that the structural ensemble of the naïve antibody has several favorable conformations and a broader free energy surface, while the matured antibody shows only one narrow and distinct minimum. Figure 1 further illustrates that the minimum in the combined PCA space observed for the matured antibody corresponds to one minimum found within the ensemble of the naïve antibody. In line with the hypothesis that the prevailing conformation in the matured ensemble is already present in the naive ensemble, rigidification during the affinity maturation shifts the probability toward the free energy minimum representing the matured loop conformation.

**Figure 2:**
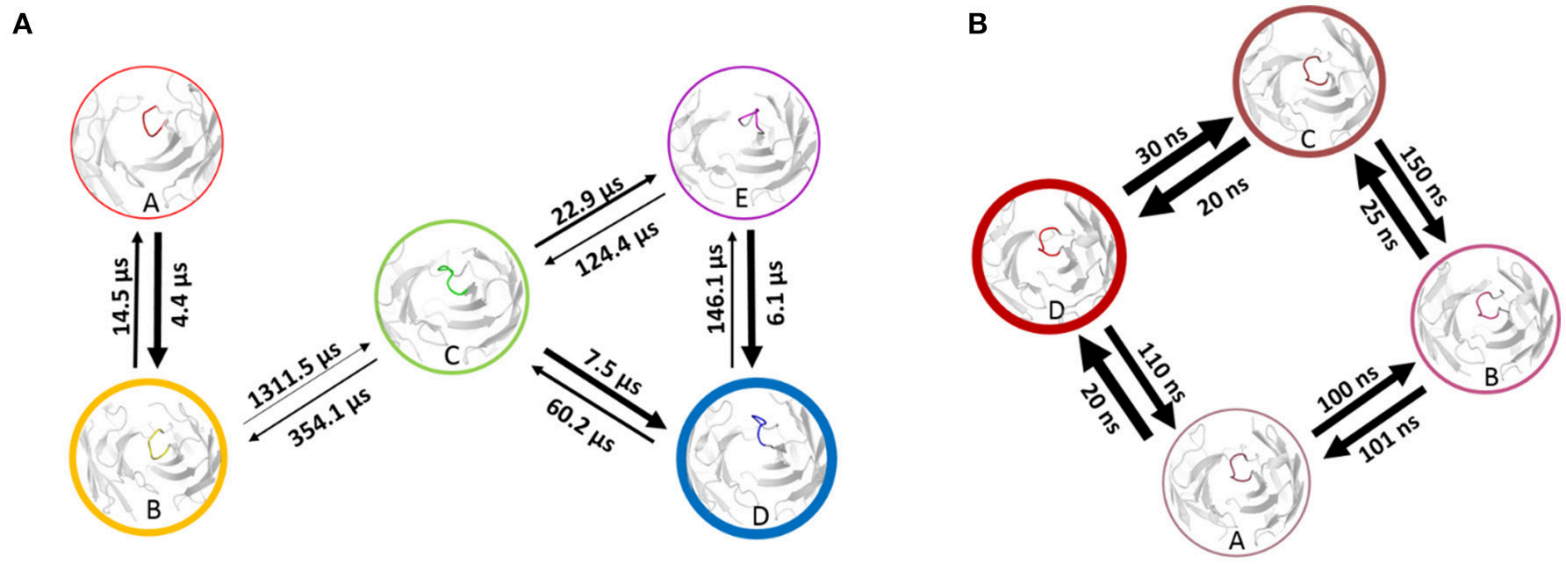
Markov-state transition timescales for the naïve **(A)** and the matured **(B)** antibody. The thickness of the circles represents state probabilities, while the width of the arrows relates to the strongly varying transition timescales.

Using the MSM scheme described in the methods section we identify 4 macrostates for the matured antibody and 5 macrostates for the naïve antibody. Transition timescales for the connected macrostates were calculated and are displayed in Figure 2. Figure S4 shows representative structures of the macrostates, highlighting the apparent differences between the diverse structural ensemble of the naïve antibody and the rigid matured antibody.

The higher flexibility of the naïve antibody is also reflected in the higher diversity of representative CDR-H3 loop structures with a RMSD ranging from 2.0 to 8.0 Å, while the matured antibody shows a RMSD for the CDR-H3 loops of only 0.5 to 2.3 Å (Figure S5).

### Affinity Maturation of 6C8 to 8B10

**Figure 3:**
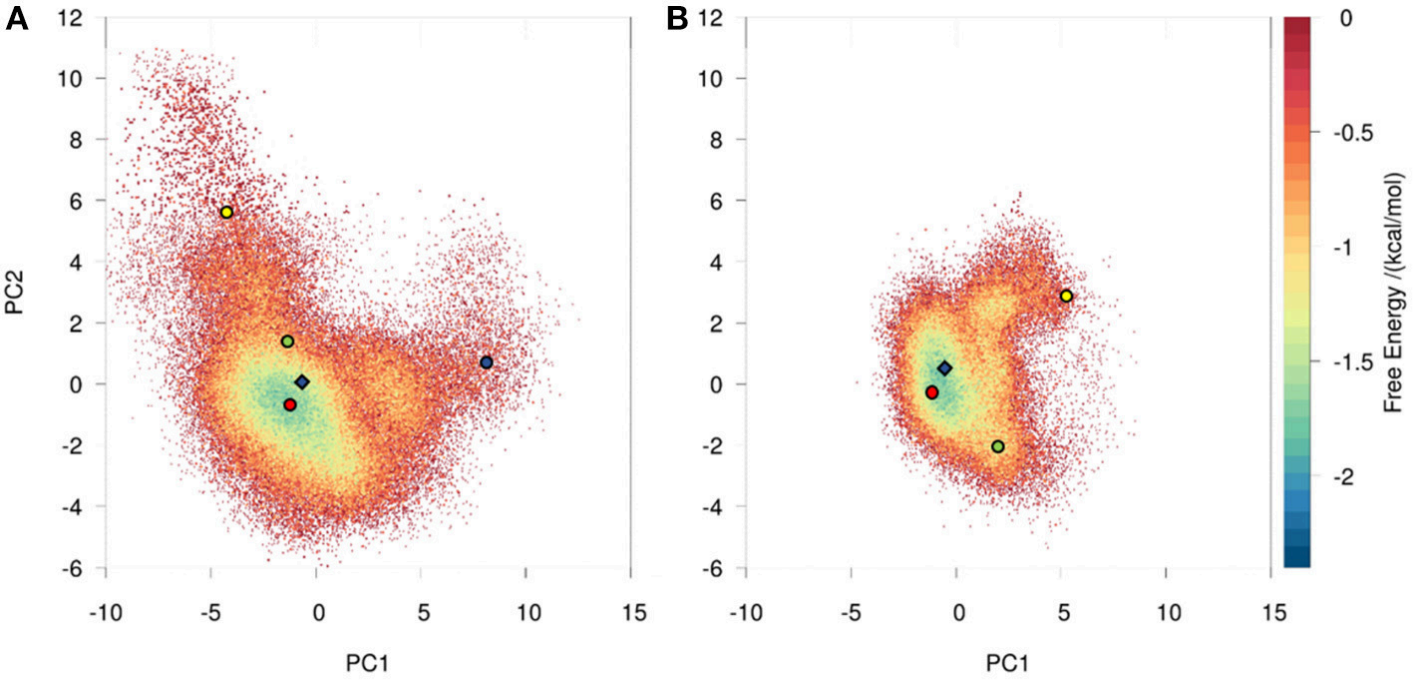
Conformational space of the 6C8 antibody **(A)** and the further matured 8B10 **(B)** antibody with the projection of the crystal structures as diamonds. The variances for the PC1 and PC2 are 55 and 14% respectively. The trajectories are projected onto the combined PCA space, illustrating the differences in conformational diversity. The PCCA+ cluster representatives are illustrated as circles color-coded according to the Markov-state model in Figure 4.

**Figure 4:**
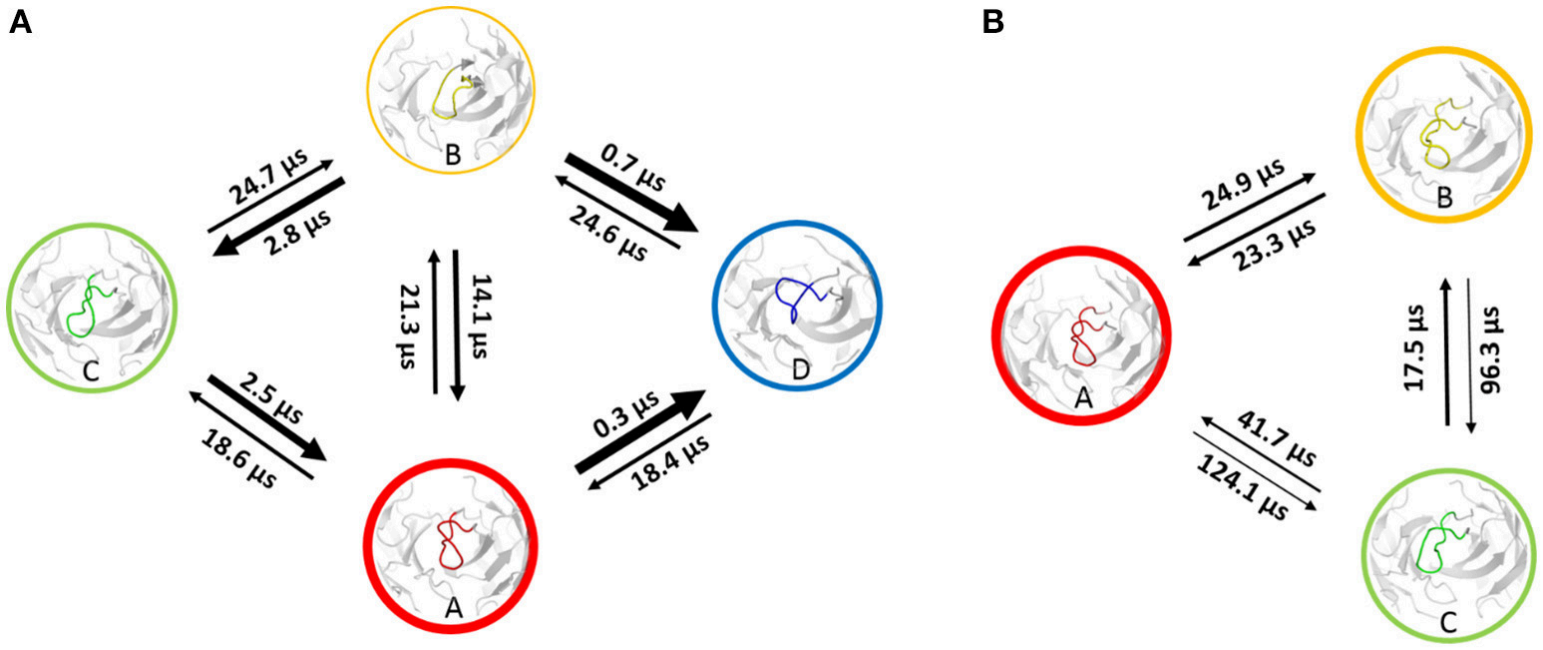
Markov-state model of the 6C8 antibody **(A)** and the further matured 8B10 antibody **(B)**. The thickness of the circles represents the state probabilities, while the width of the arrows corresponds to the transition timescales.

Using the same procedure as for the investigation of the affinity maturation of germline antibody 7G12 for clustering resulted in 99 clusters for the 6C8 antibody and led to 59 clusters for the further matured antibody 8B10 (Figure S7). Again as described in the methods section, the resulting cluster representatives were used as starting structures for 100 ns MD simulations. The projection of the trajectories onto their combined PCA space (Figure 3) illustrates that the structural ensemble of the 6C8 antibody shows a substantially higher diversity, compared to the 8B10 antibody. Estimating the kinetics we observe significantly faster transitions in 6C8 compared to the more mature 8B10. These findings are again well in line with the hypothesis that affinity maturation leads to a rigidification of the CDR-H3 loop (Figure 4). Figure S9 shows the macrostate representative structures color-coded according to the macrostates from the PCCA+ clustering. The higher flexibility of the less matured antibody is reflected in higher RMSD illustrated in Figure S10.

### Specific Antibody Fab 246 and Promiscuous Antibody Fab 249

Applying the method to the antibody pair Fab 246 and Fab 249, the projection of the resulting trajectories on their combined PCA space (Figure 6) shows several distinct minima for the specific Fab 246. The promiscuous Fab 249 shows a broader conformational space, reflecting a higher diversity of the structural ensemble (67) captured in 20 μs.

Based on clustering in the tICA coordinates, the Markov-state model was estimated to identify the kinetics of the systems (**Figure 7**). As not all the microstates are fully connected by reversible transitions, the largest connected subset of the microstates is used. The percentage of states used amounts to 90.5% of the microstates for the Fab 246 and 95.5% for the Fab 249. The high number of connected microstates emphasizes the high efficiency of the sampling as it connects most of the highly diverse starting structures. These connected sets, shown in Figure S13, are used to build the MSMs. Fuzzy clustering using PCCA+ was used to coarse-grain the 200 microstates into 3 connected macrostates for the specific system Fab 246 and 4 macrostates for the promiscuous Fab 249. Transition timescales between the connected macrostates were calculated and illustrated in **Figure 7**. The specific system Fab 246 (**Figure 7B**) shows significantly higher transition timescales compared to the promiscuous Fab 249 (**Figure 7A**). The representative structures of each macrostate are shown in Figure S15 and the RMSD values displaying the structural differences of the CDR-H3 loop of the representative structures are illustrated in Figure S16. The representative structure of state D of the Fab 246 differs from all the other structures not only kinetically, but also structurally, and is not connected to the rest of the ensemble on the timescale captured in 20 μs of classic MD simulations.

## Discussion

In this present study, we describe a protocol to characterize the structural diversity as well as thermodynamic and kinetic properties of the CDR-H3 loop using metadynamics in combination with a large number of short classic molecular dynamics simulations. While enhanced sampling methods like metadynamics allow highly efficient conformational sampling, the distortion of the underlying potential prohibits direct calculation of kinetic information. Hence, to recover the accurate kinetics of the observed structural rearrangements, we used the conformational ensemble captured with metadynamics to seed classic molecular dynamics (MD) simulations.

### Affinity Maturation of Germline Antibody 7G12

A recent study of the 7G12 antibodies with the focus on the CDR-H3 loop has shown that affinity maturation seemingly does not lead to rigidification (27). However on a substantially longer timescale, we find a significant rigidification as a consequence of maturation. Figures 1, 2 clearly show that the matured antibody displays a restricted mobility and has only one distinct minimum in the free energy surface. In general, flexibility can result from movements on different timescales (68). Small conformational changes within a shallow free energy basin can be characterized in the nanosecond to microsecond timescale, while transitions between deep minima separated by high kinetic barriers can take microseconds, milliseconds or longer (69). The timescales illustrated in Figure 2 and Figure S3 show nano- to microsecond dynamics for the matured antibody within the single distinct minimum, while the naïve antibody shows a broader free energy surface covered in the micro- to millisecond timescale. The representative structures for the resulting macrostates of the PCCA+ are shown in Figure S4. The structures of the naïve antibody (Figure S4, left) display a higher conformational diversity compared with the matured antibody (Figure S4, right). The representative structure of state A of the naïve system is similar to the crystal structure of the bound state. The representative structures of the macrostates A, B, D and E show a similar, but relocated loop conformation. The structure of the state C represents a conformation on the transition between states A and B and states D and E. The structures of the matured antibody (Figure S4, right) show only small differences in the backbone. The loop itself displays the same conformation in slightly shifted positions for all representative structures. A summary of all calculated CDR-H3 loop differences between the crystal structures and representative structures of the macrostates is shown in Figure S5. For this antibody pair we clearly observe, that affinity maturation leads to a rigidification on the captured timescale. The enhanced specificity of the matured antibody is reflected in reduced flexibility by showing a deep and narrow free energy basin. This result is also visualized in the observed timescales, which illustrate that the matured antibody stays in the same narrow and deep minimum and shows transition in the nano-second timescale.

### Affinity Maturation of 6C8 to 8B10

Adhikary et al. (30, 70) characterized sequence, structure, and plasticity of antibodies during different stages of affinity maturation and found smaller motions for the matured antibody. The characterization of the systems showed that the more specific 8B10 antibody shows reduced dynamics compared with the 6C8 antibody (Figure 3). The 6C8 antibody has a shallower and broader free energy surface compared with the affinity-matured 8B10 antibody, which shows only one deep minimum and less conformational diversity (Figure 3B). In Figure 4 the transition timescales between the different macrostates are illustrated and in both systems flexibilities on different timescales are described. The transitions in the 6C8 antibody occur fast, while the 8B10 antibody shows slower timescales for conformational rearrangements of the CDR-H3 loop. The longer transition timescales in the further matured 8B10 antibody are correlated with deeper free energy basins and higher free energy barriers (Figure S8). The representative structure of state A of the 6C8 antibody is very close to the crystal structure and is nearly identical with structure A of the 8B10 antibody. Structurally the states A, C and D of the 6C8 antibody show a similar loop conformation. The only difference can be observed in a relocation of the loop. In contrast, the representative structure of the macrostate B shows significant changes in the shape and the location of the loop compared to the structures of the states A, C and D. The system 8B10 shows high transition timescales between the minima A and C reflecting high energy barriers in the free energy surface. These slow transition timescales between the macrostates A and C can be explained by significant structural rearrangements of the CDR-H3 loop and a substantial change in the hinge angle of the loop. All this observations are also reflected in the tICA (Figure S8). The structures of state B and C show only slight differences in the loop shape itself, while the relocation of the loop, as transition to state A, dominates the structural diversity. The structural differences for the CDR-H3 loop structures of the 6C8 and the 8B10 antibody are visualized in Figure S10. The 6C8 antibody shows a broader free energy surface with lower transition timescales compared to the further matured 8B10 antibody, which illustrates higher timescales and higher free energy barriers.

### Specific Antibody Fab 246 and Promiscuous Antibody Fab 249

Also for the last system the transition timescales are significantly slower for the specific system, Fab 246, while the transitions between conformational states of the Fab 249 occur much faster (**Figure 7**). The representative CDR-H3 loop macrostate structures show higher deviations in the kink region of the loop than in the loop itself. The representative structure of the state D, which shows no reversible transitions to the other macrostates represents the structure with the highest differences in loop and kink region. The structural differences of the CDR-H3 loops are calculated by using two-dimensional RMSD to compare the structures among each other (Figure S16). Fab 249 shows the highest structural changes in the loops itself, which means that additional to the relocation of the loop, reshaping of the CDR-H3 loop for all four macrostate representatives can be observed. The promiscuous system shows a slightly broader free energy surface (Figure 6) compared with the specific system. The main difference between this pair of antibodies, illustrated in Figure 6, is that the specific antibody shows deeper and narrower free energy minima, compared with the promiscuous system, which shows a shallow free energy surface. Due to the fact, that the specific antibody Fab 246 displays deeper minima in free energy and higher kinetic barriers, more sampling time would be required to have fully connected initial microstates and to be able to calculate transition rates for all four states, including state D in Figure S14 (cf. microstates in Figure S13). The specific antibody Fab 246 shows one fast transition between the macrostates A and B, because they are located in the same minimum, while the transitions to macrostate C occur on a significantly longer timescale. The same hypothesis as discussed in the previous examples, is strengthened here, that higher specificity is connected with deeper and more distinct free energy basins, which is reflected in the longer transition timescales and higher free energy barriers. We observe also here that enhanced specificity results in reduced flexibility in the CDR-H3 loop.

**Figure 5:**
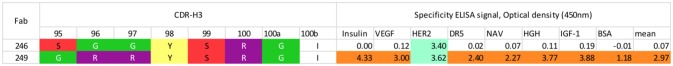
CDR loop sequences (left) and affinities (right) of Fab 246 and Fab 249 CDR-H3 loop. The affinities of the two Fabs were tested against a set of eight antigens (Insulin, VEGF-Vascular Endothelial Growth Factor, HER2-Human Epidermal Growth Factor Receptor 2, DR5-Death Receptor 5, NAV-Neutravidin, HGH-Human Growth Hormone, IGF-1-Insulinlike Growth Factor 1, BSA-Bovine Serum Albumin) (31). The numbering of the CDR residues is according to the nomenclature of Kabat et al. (71).

The results found in this study highlight that sequence-based or static structural information alone might not be sufficient to understand and describe antibody binding properties as, e.g., specificity and promiscuity. Long timescale dynamics from enhanced and classic MD simulations complement experimental structural information with reliable estimations of conformational preferences and state probabilities. A sequence based study on multispecificity focusing on the CDR-H3 loop, shows that the introduction of arginine enhances the promiscuity of antibodies (Figure 5, Figure S12) (31). This finding seems to be controversial since polar and charged residues are often found to contribute to specificity by establishing electrostatic and hydrogen-bonding interactions, which rather enhances the complex stability (72). In our simulations we observe that the two neighboring arginine residues next to each other in the Fab 249 show repulsive behavior and thereby increase the flexibility of the CDR-H3 loop. Also the role of tyrosine is still controversial. On the one hand, due to its amphipathic and aromatic character it can make various different interactions and is also known as a “sticky” residue (73). On the other hand, it has been shown that specificity and affinity is enhanced in the antigen recognition process by introducing tyrosine residues in combination with small residues like glycine and serine (31). The higher content of glycine residues in the specific antibody is highly surprising, since glycine is usually known to increase flexibility (74). However, the position of the glycine residues in the loops and the influence of other neighboring amino acids may cause the observed rigidification.

**Figure 6:**
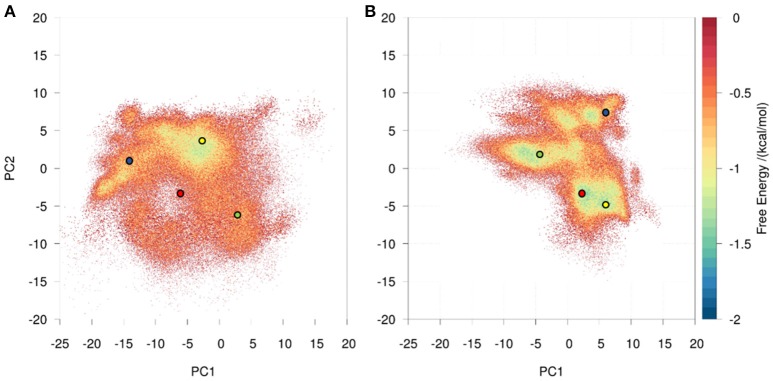
Conformational space of the promiscuous Fab 249 **(A)** and the specific Fab 246 **(B)** antibody. Both 20 μs trajectories are projected onto the combined PCA space, clearly showing that the specific antibody has more distinct and narrower free energy basins, while the promiscuous system shows a shallow and broad free energy surface. The PCCA+ cluster representatives are illustrated as circles color-coded according to the Markov-state model in Figure 7. The variances for the PC1 and PC2 are 58 and 13% respectively.

**Figure 7:**
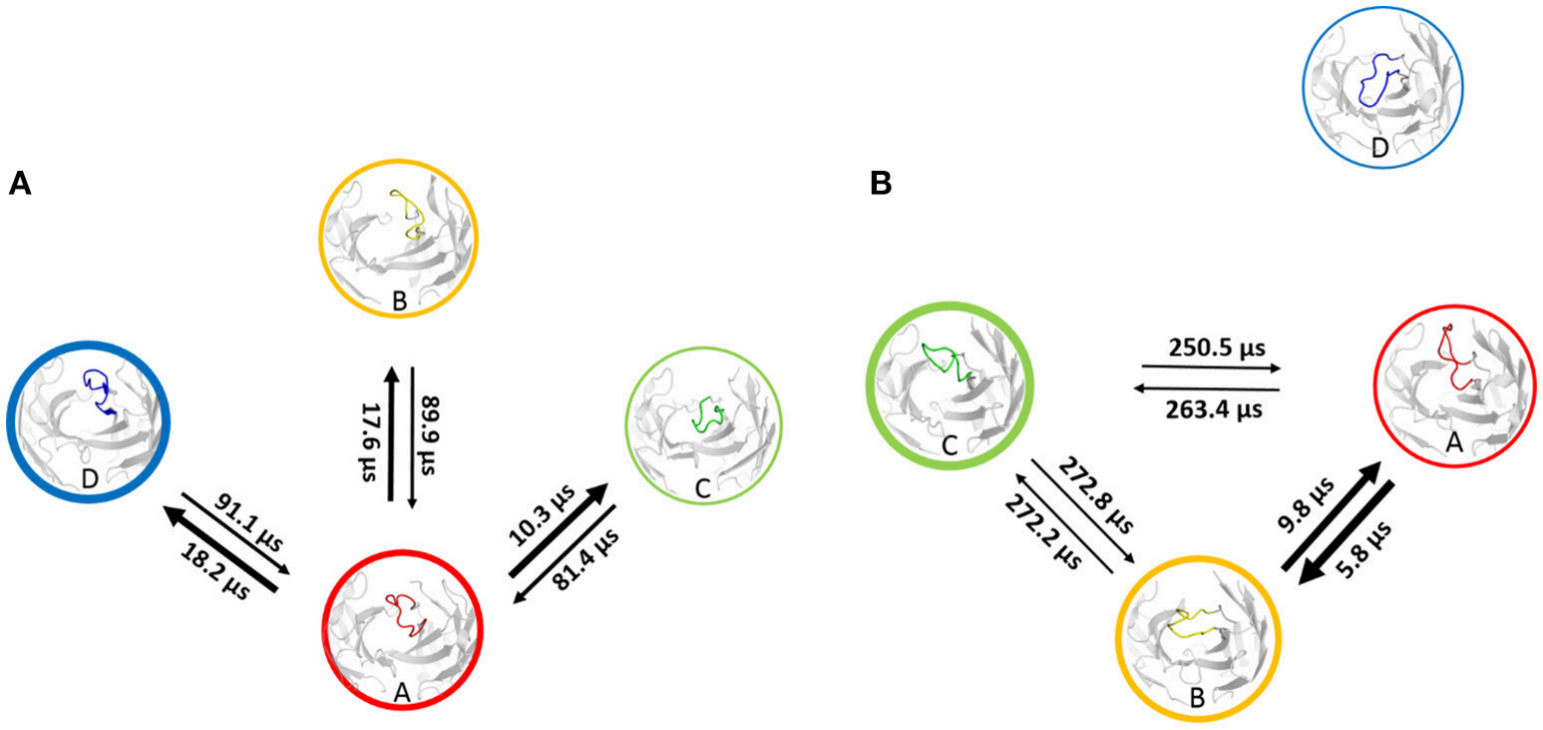
Transition timescales based on a Markov-state model of the promiscuous Fab 249 **(A)** and the specific Fab 246 **(B)** antibody. Fab 246 shows significantly higher transition timescales, which indicate higher free energy barriers, compared with the Fab 249.

In contrast to this sequence based view, we aim at understanding promiscuity as a structural property governed by dynamics. CDR-H3 loop structures are very difficult to predict because their structures cannot be compared with any other protein loops found in databases and the CDR-H3 loop is known to be the most flexible (3, 11, 75, 76). Thus, sampling efficiency plays a key role in linking specificity and rigidity of the CDR-H3 loop as many different conformations have to be covered. For two examples of antibodies before and after the affinity maturation crystal structures are available and used as starting points for simulations. The third antibody system is modeled using the program RosettaAntibody. The reliability of a structural prediction of the CDR-H3 loop decreases with increasing number of residues in the CDR-H3 loop (11, 13). To tackle these difficulties, we used the kinematic loop closure algorithm (37) additional to the RosettaAntibody to diversify the starting structures of the CDR-H3 loop. Classic MD simulations allow to consider not only a static antibody structure, but also to characterize the dynamic properties and to describe the CDR-H3 loop as conformational ensemble. Enhanced sampling techniques are essential to overcome high energy barriers of the potential energy surface and to more exhaustively describe and characterize the antibody CDR H3-loop. Metadynamics is only one of many solutions to face the sampling problem (77, 78). We employ this technique to gather structures that we use as starting points to seed a large number of short classic MD simulations (79). To extract kinetic information from these shorter MD simulations, a Markov-state model was built that enables to combine the simulations into a statistical model (64). Figures 2, 4, 7 show our results for long-timescale molecular dynamics simulation data obtained from short classic MD simulations. In all three systems the antibodies with higher specificity display higher kinetic barriers and longer transition timescales according to the estimated free energy surface. This is in line with the hypothesis that antibodies become more rigid during affinity maturation (67). The broad free energy basins of the promiscuous systems in combination with lower kinetic barriers allow to adopt more conformational states. In contrast, the rigidity of the specific systems hinders binding of more diverse antigens (67). Different studies have been focusing on the question whether conformational flexibility in germline antibodies is promoted by their native sequence (28, 76). *In silico* approaches to design antibodies mimicking *in vivo* affinity maturation are in line with our observations that specific antibodies have a reduced conformational diversity (28, 76) Somatic hypermutations contribute to the affinity maturation process by modifying the shape of the paratope, restricting the mobility of paratope residues and improving complementarity to the epitope (80). Descriptions of affinity maturation pathways in combination with long timescale molecular dynamics simulations lead to the conclusion that an increase in specificity is directly correlated with a rigidification of the initially flexible CDR-H3 loop (4). However, the rigidification of the antibody might be only one of the many mechanisms involved in the affinity maturation process. Additional salt bridges and hydrogen bonds as well as an improved shape complementarity specific to the antigen might also contribute in the affinity maturation process (81).

## Conclusion

With the present study, we characterize the CDR-H3 loop in the binding interface of three pairs of antibodies and link specificity with rigidity. Antibodies with and without available structural information were analyzed to show that an approach starting from sequence information alone can be applied to characterize antibody CDR-H3 loops as well. We used the program RosettaAntibody in combination with additional cycles of the kinematic loop closure algorithm to predict antibody structures, differing in the CDR-H3 loop. To overcome the limitations of classic molecular dynamics simulations, we performed metadynamics simulations and observed a broader and more efficient exploration of the conformational space. The newly captured ensemble was clustered using a hierarchical clustering algorithm. The resulting representative structures are used as seed structures for a large number of shorter molecular dynamics simulations to obtain kinetic information. We coarse-grained the resulting conformations to macrostates and calculated transition timescales based on Markov-state models for each antibody pair, respectively. To sum up, we observed that a higher specificity correlates with distinct free energy basins and slower transition timescales. The promiscuous or naïve systems show shallow free energy basins and the transitions between different states occur much faster than in the specific or matured antibodies. With our protocol we observe that within these three pairs of antibodies, the antibodies binding more antigens are more flexible and have shallower free energy surfaces, which makes different conformational states accessible on a shorter timescale. Even though the methods applied are computationally quite demanding and thus not applicable as a high-throughput screening technique, advances in GPU computing allow for this state-of-the art analyses to be applied easily to antibodies of high significance. Possible applications of our approach might reach from improvements for protein-protein docking of antibodies considering conformational ensembles as far as fine tuning of therapeutic antibodies in terms of their multispecificity.

## Author Contributions

All authors listed have made a substantial, direct and intellectual contribution to the work, and approved it for publication.

### Conflict of Interest Statement

The authors declare that the research was conducted in the absence of any commercial or financial relationships that could be construed as a potential conflict of interest.

## Abbreviations

CDR: Complementary determining region
Fab: Antigen binding fragment
Fv: Variable domain
KIC: Kinematic loop closure algorithm
MD: Molecular dynamics
MSM: Markov-state model
PCA: Principle component analysis
PCCA: Perron-cluster cluster analysis
RMSD: Root mean square deviation
tICA: Time-lagged independent component analysis.
