# Supplementary material for: Characterizing the Diversity of the CDR-H3 Loop Conformational Ensembles in Relationship to Antibody Binding Properties
Source: Front Immunol. 2019 Jan 7;9:3065. doi: 10.3389/fimmu.2018.03065 (PMC6330313; doi:10.3389/fimmu.2018.03065)
Supplement: Supplementary file 1 [file Data_Sheet_1.docx]

SuppORtING INformation

**Affinity maturation of the germline antibody 7G12**

| 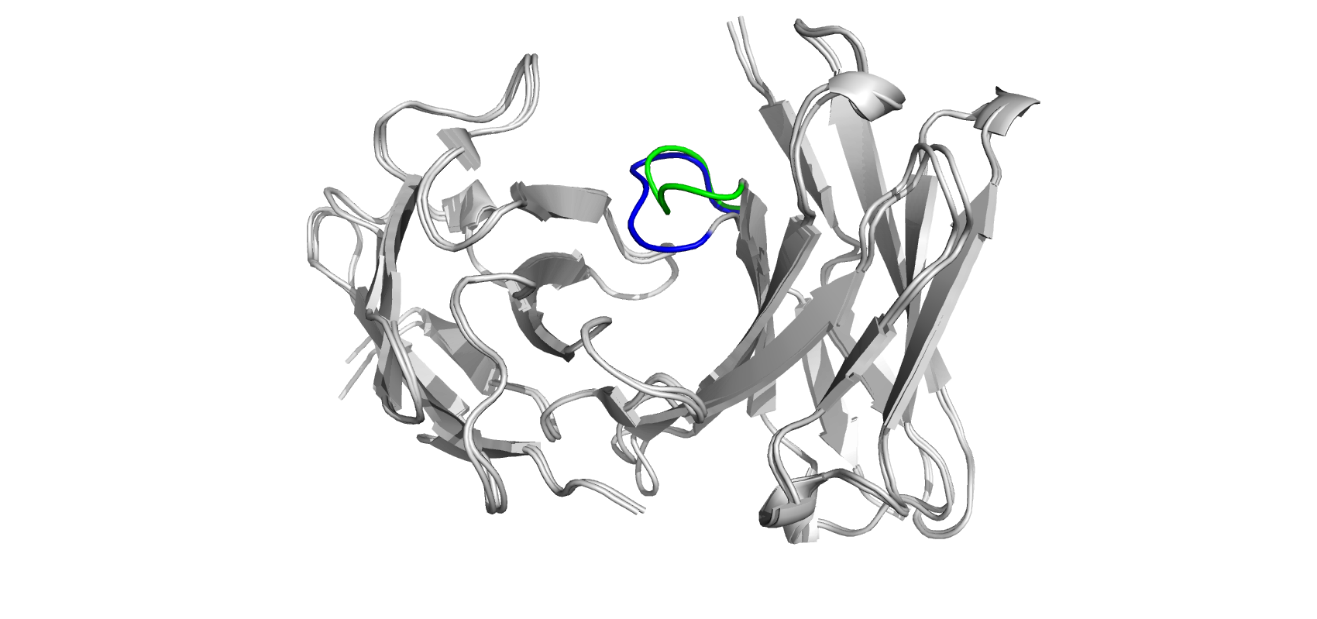 | 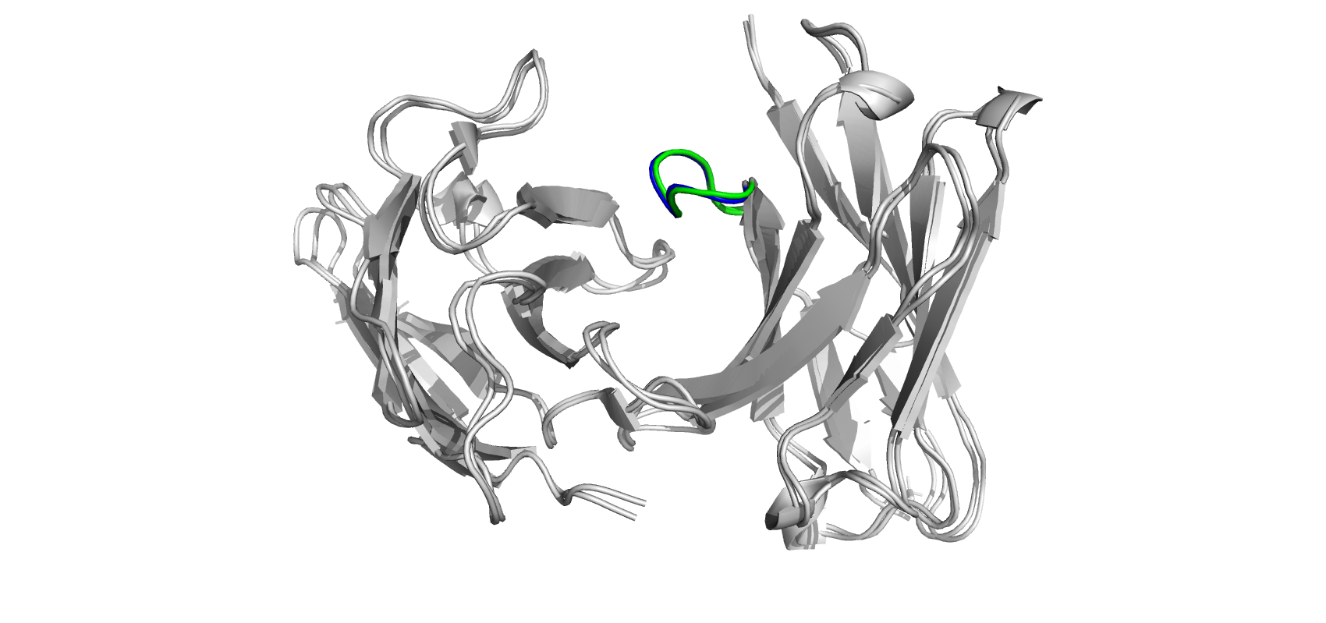 |
| --- | --- |

Figure S1: The structures of the germline antibody 7G12 are shown on the left, the structures of the matured antibody are shown on the right. The CDR-H3 loops are colored in green for the bound states (shown and simulated without antigen present) and in blue for the free states.


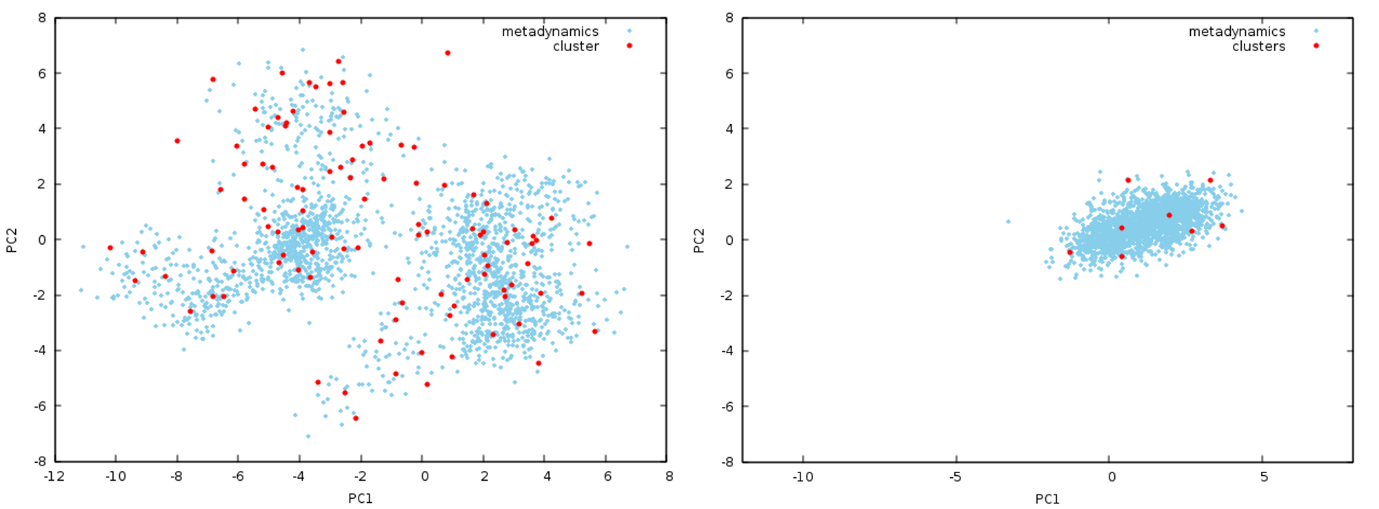


Figure S2: Cartesian PCA based on the the Cα coordinates of a combined 2 µs metadynamics trajectory of the naïve (left) and the mature (right) antibody. Projection of the cluster representatives (red), which are used as starting structures for short MD simulations.


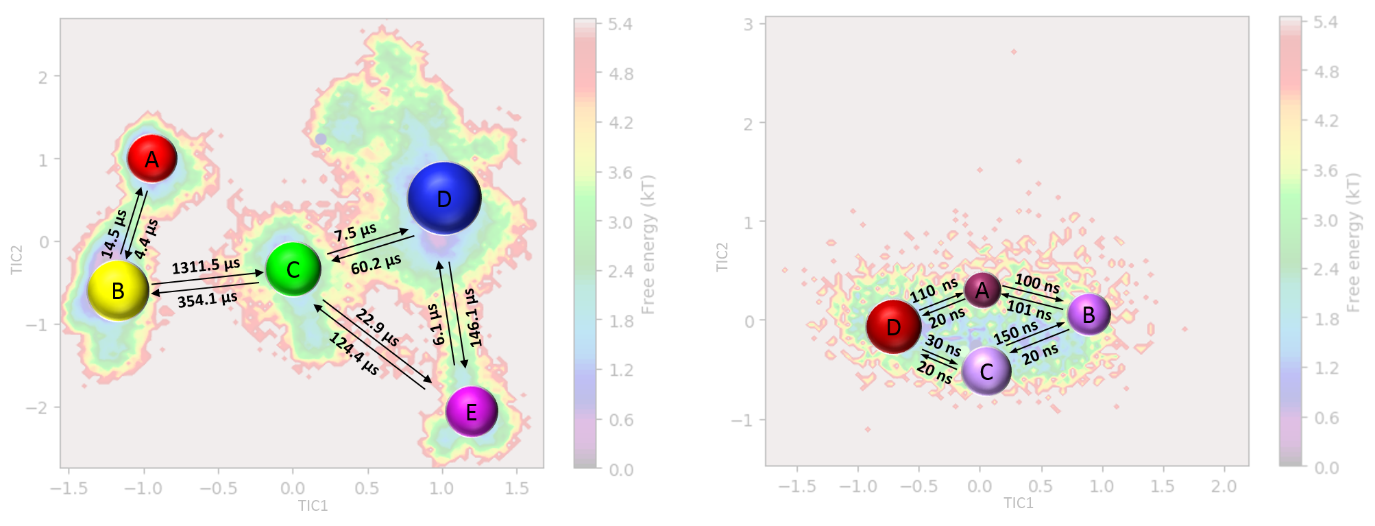


Figure S3: Transition timescales for the naïve 7G12 antibody (left) and the matured antibody (right) with the calculated tICA in the background. The size of the colored spheres correlates with the calculated probability of the macrostates.


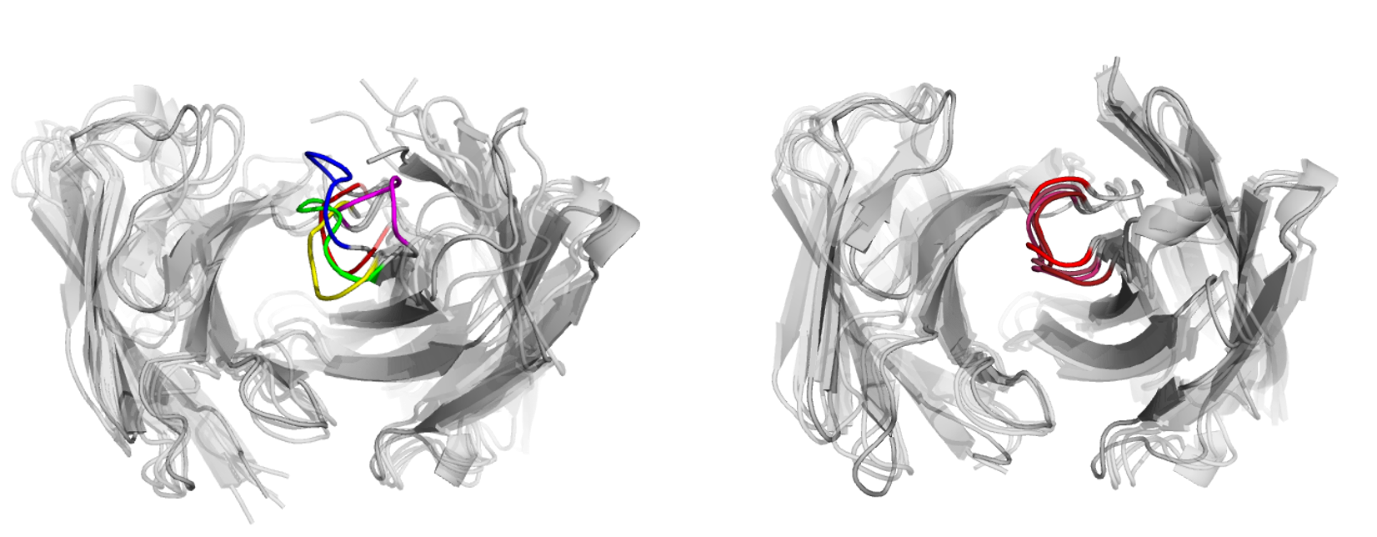


Figure S4: Structures representing the macrostates for the naïve 7G12 antibody (left) and the matured antibody (right) are compared. The color of the CDR-H3 loop corresponds to the coloring of SI Figure S3, red for state A, yellow for state B, green for state C, blue for state D and magenta for state E. The matured antibody shows very similar structures for the CDR-H3 loop and correspond to the minimum A of the naïve antibody.

Figure S5: 2D RMSD plot of the CDR-H3 loops (Cα) of the crystal structures and the representatives of the macrostates for the naïve and the matured 7G12 antibody. The values are given in Å and were calculated by aligning on the whole Fv.

## Affinity maturation of 6C8 to 8B10 antibody


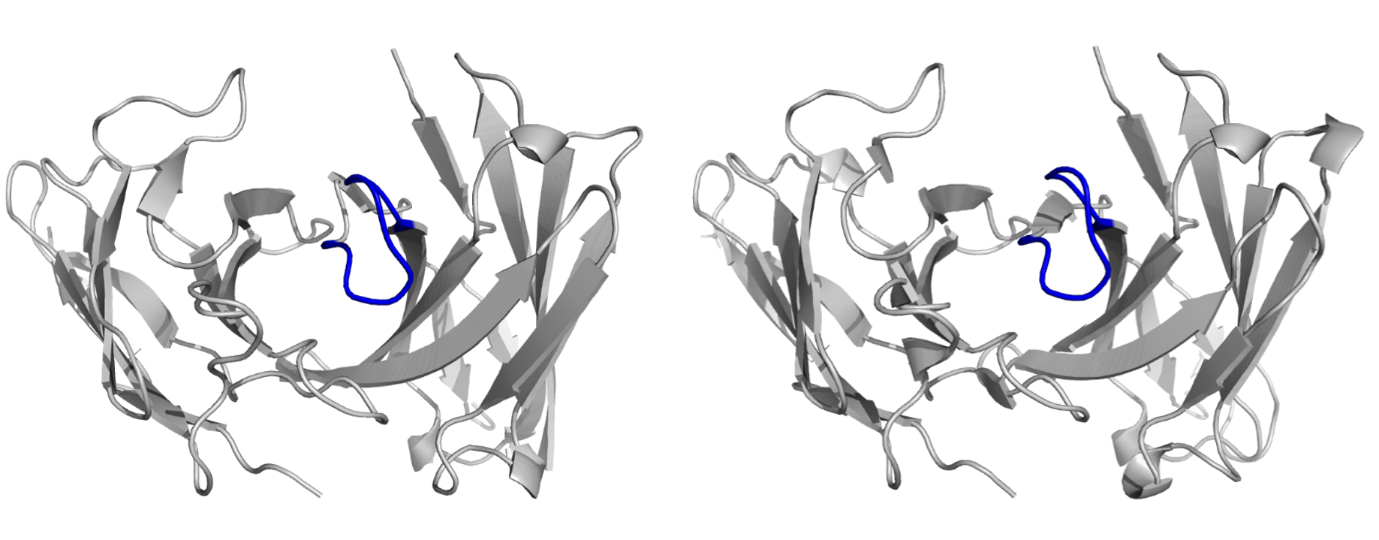


Figure S6: Crystal structures of the 6C8 (left, PDB code 4NJA) and 8B10 (right, PDB code 4NJ9) antibodies.


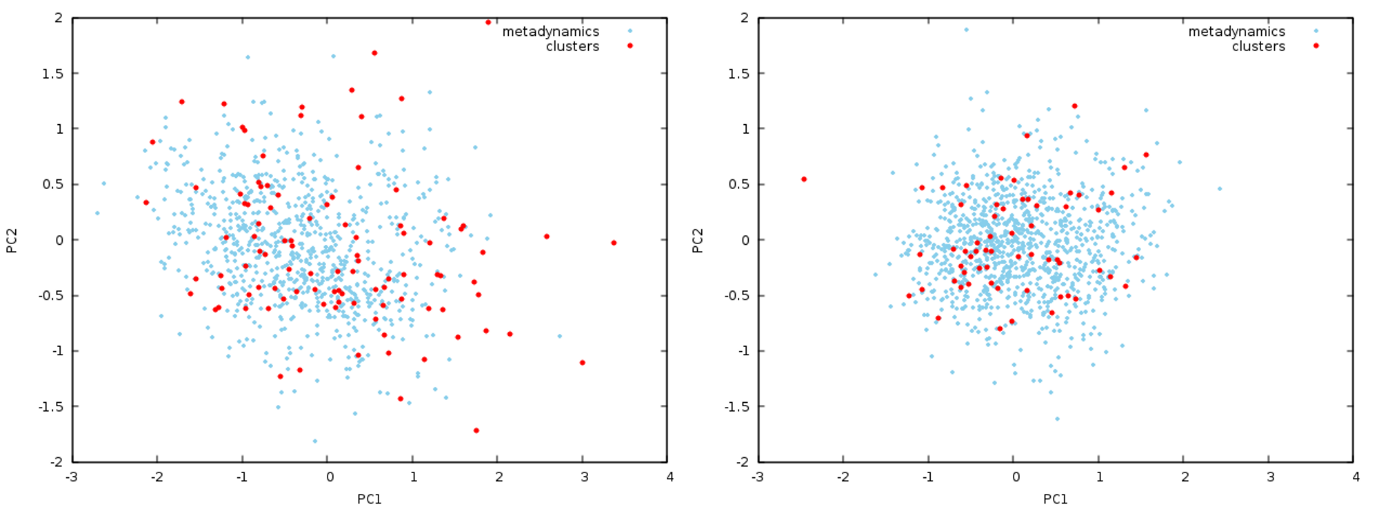


Figure S7: Cartesian PCA of the Cα coordinates of a 1 µs metadynamics trajectory of the 6C8 and the 8B10 antibody with the projected cluster representatives (red), which were used as starting structures for short MD simulations.


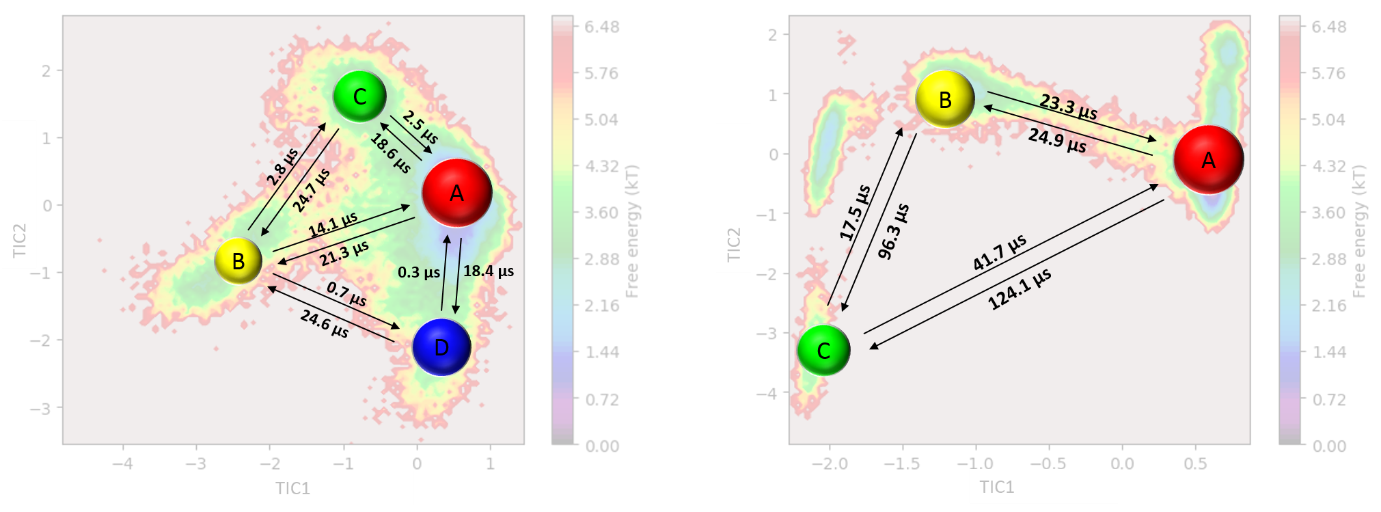


Figure 8: Estimated free energy surface based on tICA of Cα coordinates including reversible transition timescales for the 6C8 (left)and the further matured 8B10 antibody (right).


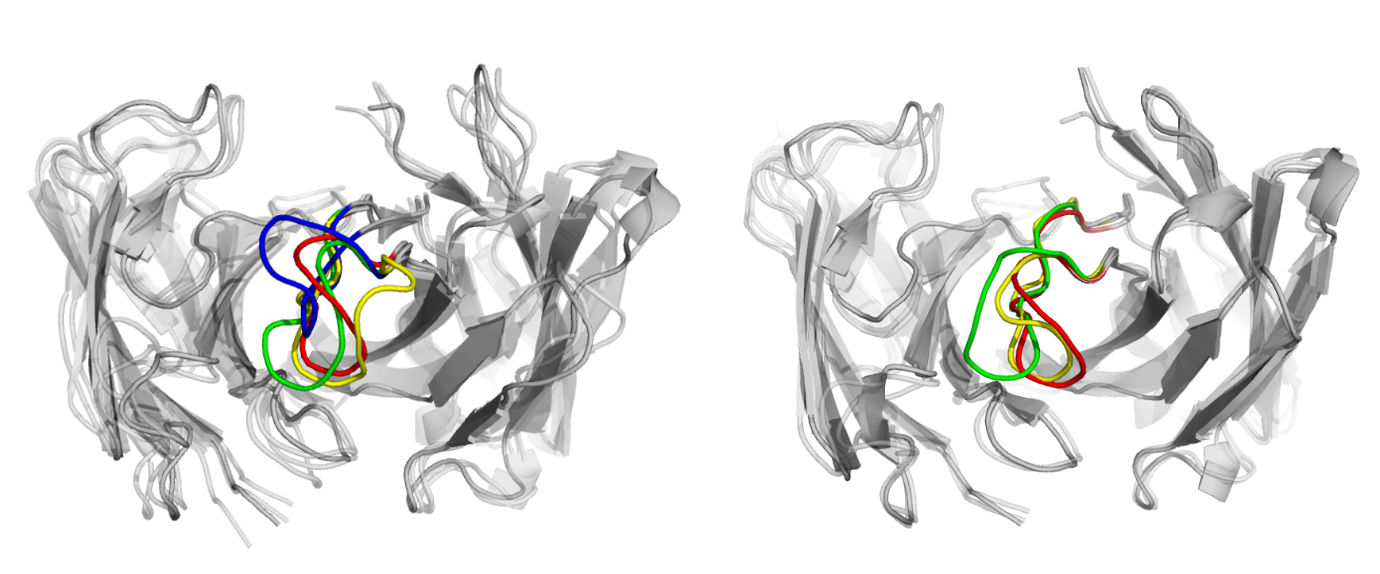


Figure S9: Representative macrostate structure for the 6C8 antibody (left) and the further matured 8B10 antibody (right).

Figure S10: 2D RMSD plots of the CDR-H3 loops (Cα) calculated by aligning the whole Fv with the values in Å of the crystal structures with the corresponding representatives of the macrostates for the 6C8 and the 8B10 antibody.

## Specific antibody Fab 246 and promiscuous antibody Fab 249


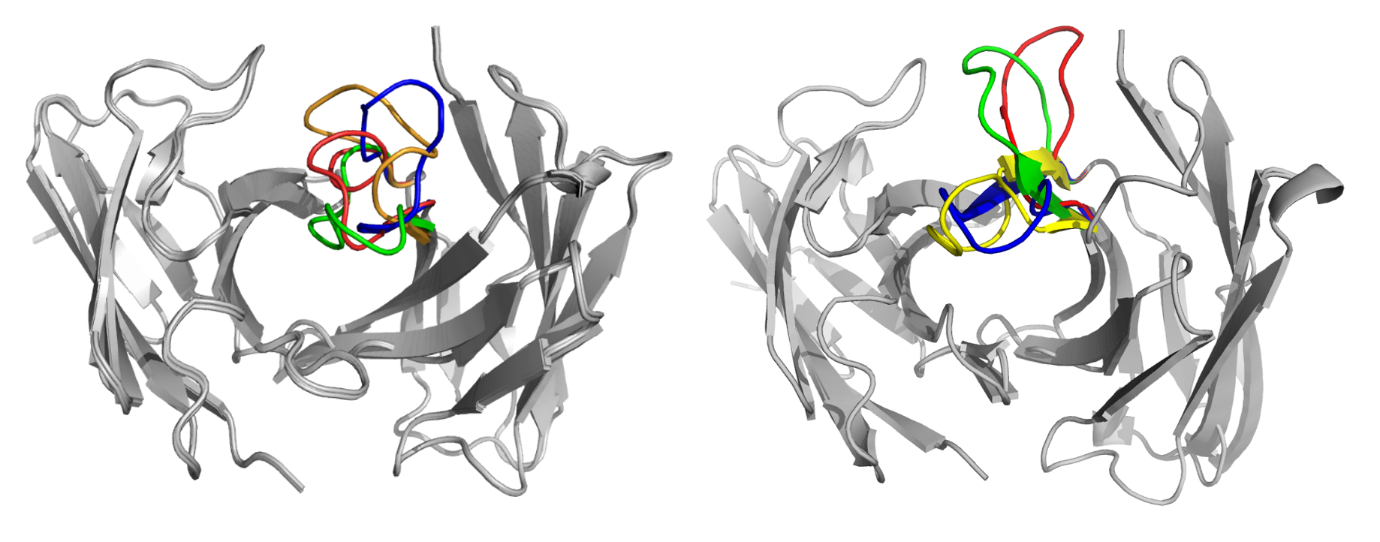


Figure S11: Four modeled structures of the promiscuous system (Fab 249, left) and the specific (Fab 246, right) differing in the structure of CDR-H3 loop.

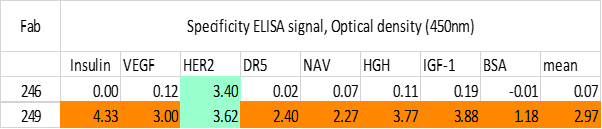


Figure S12: CDR loop sequences (top) and affinities (bottom) of Fab 246 and Fab 249^20^. The affinities of the two Fabs were tested against a set of eight antigens (Insulin, VEGF-Vascular Endothelial Growth Factor, HER2-Human Epidermal Growth Factor Receptor 2, DR5-Death Receptor 5, NAV-Neutravidin, HGH-Human Growth Hormone, IGF-1-Insulinlike Growth Factor 1, BSA-Bovine Serum Albumin).^20^ The numbering of the CDR residues is according to the nomenclature of Kabat et al.^36^

Table S1: Template structures which were used for the structure modeling of the CDR-loops of Fab 246 and Fab249.

| Fab 246 | PDB Codes | Fab 249 | PDB Codes |
| --- | --- | --- | --- |
| CDR-L1 | 1FVC | CDR-L1 | 1FVC |
| CDR-L2 | 3GRW | CDR-L2 | 1TZH |
| CDR-L3 | 1H3P | CDR-L3 | 1H3P |
| CDR-H1 | 2FJF | CDR-H1 | 2HFG |
| CDR-H2 | 1FVC | CDR-H2 | 1FVC |
| CDR-H3 | 1A3L | CDR-H3 | 1JN6 |


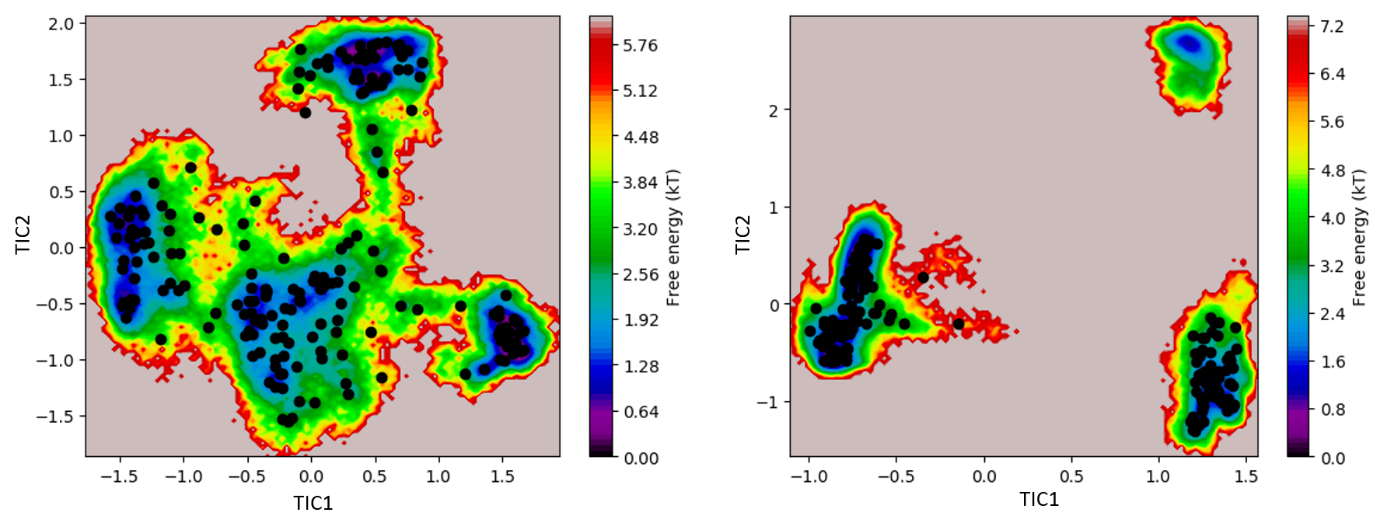


Figure S13: Largest sets of connected states for the promiscuous Fab 249 (left) and the specific Fab 246 (right) are shown as black points.


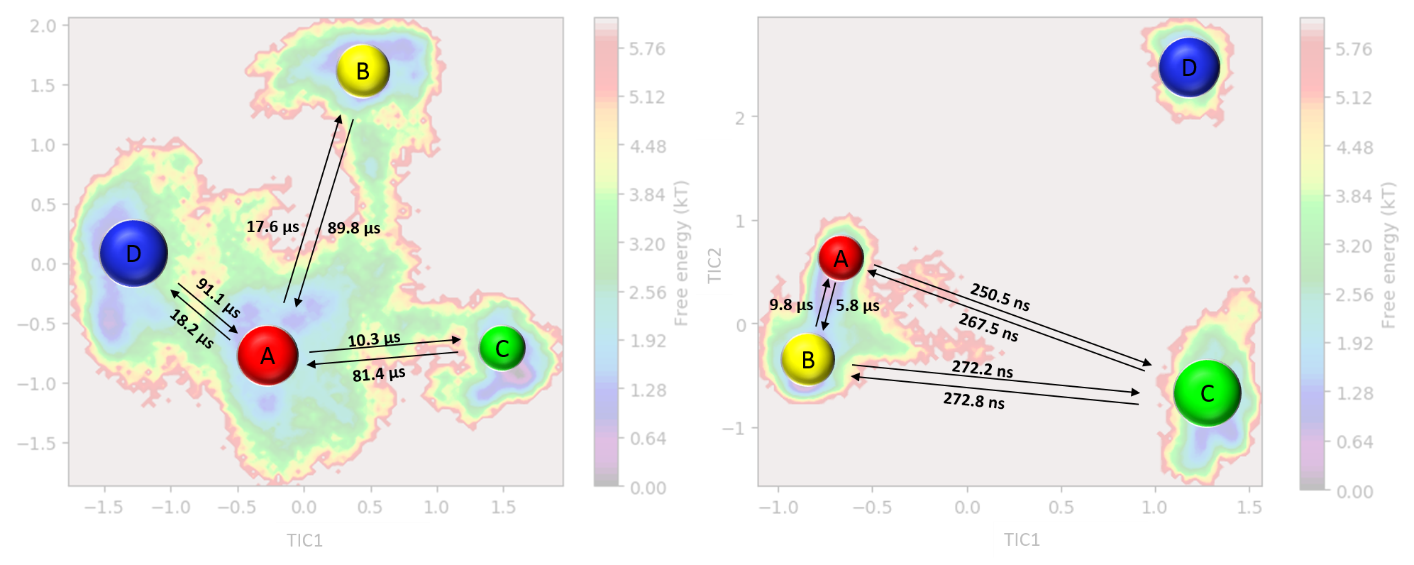


Figure S14: Estimated free energy surface based on tICA Cα coordinates including reversible transition timescales for the promiscuous Fab 249 (left) and for the specific Fab 246 (right).

| 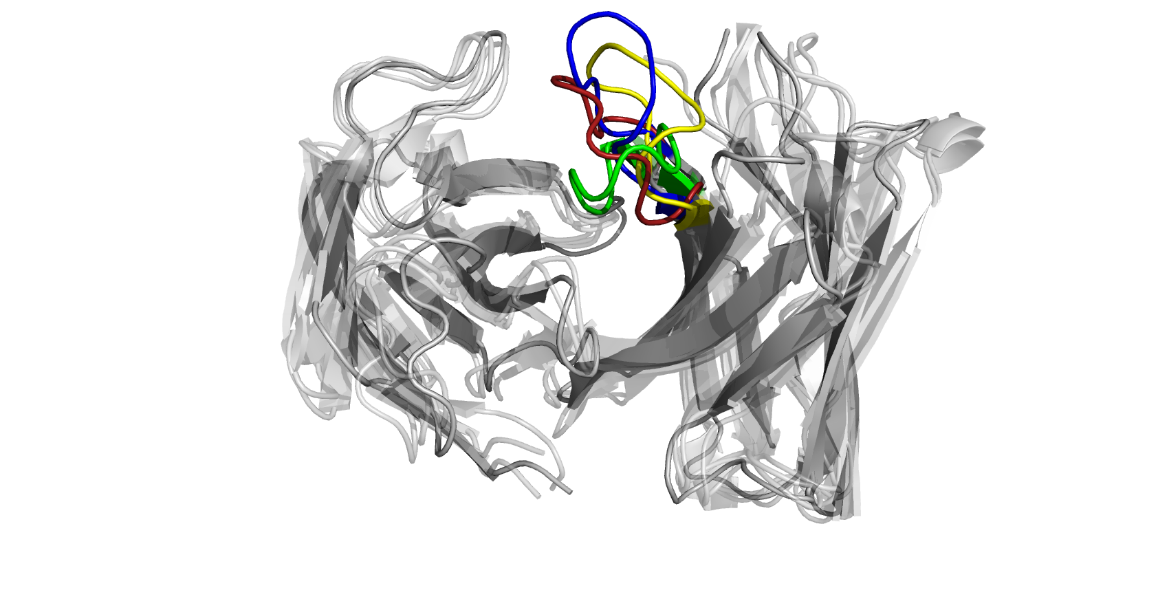 | 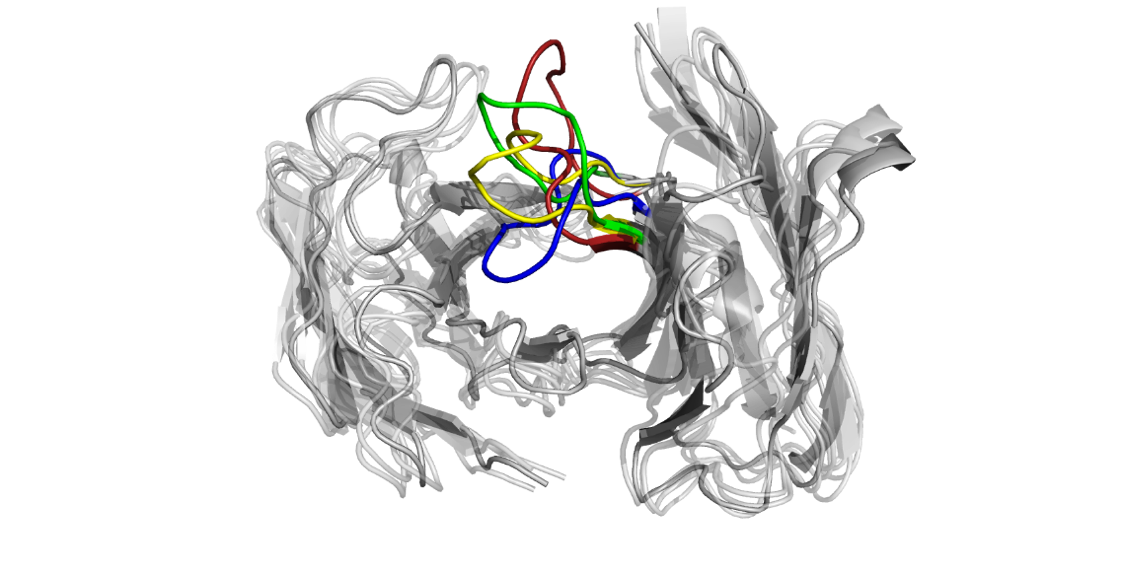 |
| --- | --- |

Figure S15: Structures representing the macrostates in the tICA space for the promiscuous Fab 249 (left) and the specific Fab 246 (right) antibody. The representative of state A is colored in red, of state B is colored in yellow, of state C is colored in green and of state D is colored in blue.

.

Figure S16: 2D RMSD plots of the CDR-H3 loops (Cα) calculated by aligning the whole Fv with the values in Å of the 4 representative structures of the macrostates of the Fab 249 (left) and the Fab 246 (right).
